# The Social Contract at Risk: COVID-19 Misinformation in South Africa

**DOI:** 10.4102/jamba.v16i1.1630

**Published:** 2024-10-11

**Authors:** Wouter H. Kruger, Ivan Henrico, Hendrik A.P. Smit

**Affiliations:** 1 Department of Military Geography, Faculty of Military Science, Stellenbosch University, Saldanha, South Africa

**Keywords:** false information, misinformation, disinformation, fake news, COVID-19, pandemic, social contract, South Africa

## Abstract

Technological advancements and the ubiquity of digital platforms have accelerated the spread of false information, undermining governance and social harmony. Despite its significance, there remains no academic consensus on a taxonomy for the various manifestations of false information. This study addresses this gap and examines the complex societal implications of misinformation. The research identifies motives behind the spread of false information and assesses its impact on governance and social contracts, with a focus on South Africa during the early phase of the coronavirus disease 2019 (COVID-19) pandemic. A qualitative approach was followed, using documentary research and secondary analysis of qualitative data. While global trends in misinformation were initially explored, the study primarily focused on South Africa, covering the period from the first confirmed COVID-19 case in Wuhan in December 2019 to 5 March 2021, one year after South Africa’s first reported case. A literature review was conducted to examine the origins and spread of misinformation, including government measures and public responses. The findings revealed that power and greed are primary drivers of misinformation, with entrenched worldviews, scepticism towards authority, and a lack of critical evaluation skills intensifying the issue. The spread of misinformation had significant impacts on governmental crisis management and social cohesion in South Africa.

**Contribution:**

This study contributes to understanding misinformation’s societal impact and provides a framework for future empirical studies on crisis management and government-citizen relations. It aligns with the journal’s focus on contemporary challenges in information dissemination.

## Introduction

The start of 2019 saw the world being thrown into crisis because of the coronavirus disease 2019 (COVID-19) pandemic. By 05 March 2021, COVID-19 had infected 115 314 661 people globally and killed 2 564 723 (World Health Organization 2021). South Africa had accumulated more than 1 520 200 positive cases and suffered more than 50 600 deaths (South African Department of Health 2021). Most countries had been affected, and all governments were compelled to implement measures to either prevent the virus from entering their territories or mitigate the effect of the virus once it had reached their shores. The new media age has seen an exponential rise in false information on global networks, and South Africa’s diverse cultural, ethnic, political and historical makeup has the potential to exacerbate the impact of its exposure to information that is intended to create divides (Bradshaw & Howard 2018; Wasserman 2020).

The response of the South African government to the pandemic and the public’s acceptance of it must be viewed through the lens of the social contract theory. Jean-Jacques Rousseau, in his seminal work ‘The Social Contract’ published in 1762, argued that individuals consent to surrender certain freedoms and submit to the authority of the state in exchange for protection of their remaining rights (Rousseau 2012). This theory underscores the relationship between the state and its citizens, as well as among citizens themselves. According to Rousseau, legitimate political authority depends on this social contract agreed upon by all citizens for their mutual benefit. During crises, such as the COVID-19 pandemic, the social contract’s stability is crucial. Cinelli et al. (2020) noted that a weakened social contract in times of crisis could have dire consequences for social cohesion. The proliferation of false information during the pandemic threatens the stability and security implied by the social contract (Peters, Jandrić & Mclaren 2020).

Previous research highlights a significant rise in misinformation during health crises, which exacerbates public fear and mistrust (Pennycook et al. 2020; Tasnim, Hossain & Mazumder 2020). Wasserman (2020) and Mutsvairo and Bebawi (2019) have explored the role of social media in spreading false information in South Africa, emphasising the unique challenges posed by the country’s sociopolitical context. Additionally, Cinelli et al. (2020) analysed misinformation during the COVID-19 pandemic in Italy, emphasising the importance of empirical data in understanding its impact. Kouzy et al. (2020) examined the collective response to misinformation and its effects on public health measures.

This research aims to determine the prevalence of false information in South Africa during the first year of the COVID-19 pandemic. Despite widespread acknowledgment of increased misinformation since the pandemic’s onset (Pennycook et al. 2020; Tasnim et al. 2020), academic exploration of this phenomenon remains limited. Understanding the extent of false information dissemination and its impact on South African society and governance is essential. This study also investigates the types of individuals or entities spreading misinformation, the nature of the false information and the motivations behind such actions. This research is significant because it contributes to the existing knowledge on misinformation and its effects on social cohesion, trust in government and the social contract during a global crisis.

Moreover, this article provides several key contributions essential for understanding and managing misinformation within the context of the COVID-19 pandemic in South Africa. Firstly, it offers a detailed description of the misinformation phenomenon, providing a foundation for a nuanced understanding within a specific geopolitical context. Secondly, the article draws on empirically measures about the prevalence of false information during the pandemic’s initial year, offering concrete data on the issue’s magnitude (Cinelli et al. 2020). A critical aspect of the research is its analysis of the impact of misinformation on governmental effectiveness during crises (Kouzy et al. 2020). By drawing on the theoretical foundation of the social contract, this research illustrates how misinformation dissemination can be seen as a breach of this fundamental sociopolitical agreement. Additionally, the study explores the diverse motives behind misinformation, from power and greed to uninformed citizen behaviours, providing insights into the various factors driving the spread of false narratives. These contributions highlight the complex intersections between misinformation, governance and societal responsibilities during times of crisis.

## Material and methods

The sources covered in the literature review allowed a comprehensive analysis of false information and its effect on people and governments. The present challenge, however, is that the diffusion of false information, which has evolved over the years from first being a word-of-mouth exercise, then becoming a paper-dependent venture (pamphlets, newspapers) and later being spread via radio and television, has in the present day become an accessible avenue for anyone with a computer or smartphone. The World Wide Web has changed how people access and interpret information, while relentless technological advancements are at the same time accelerating the pace and magnitude of information sharing. These developments have metaphorically prepared the soil for false information to flourish, and it is no wonder that influential international organisations like the World Economic Forum (WEF) warn that the threat of massive digital disinformation is a risk to humankind (World Economic Forum 2013).

This study effort followed a qualitative approach or strategy, which involves research that is focussed on words rather than quantities when it comes to collecting and analysing data. It also favours inductive reasoning over deductive reasoning, adopts an interpretivist model rather than a positivist model and posits the view that individuals create their ever-changing reality (constructivism rather than objectivism). The study’s research design needed to be established as it serves as the framework or plan that guides the collection and analysis of data. The research design adopted here is documentary research or secondary analysis of qualitative data. This is a non-traditional qualitative approach, as it does not follow the traditional research phases that are characteristic of efforts such as narrative and counter-narrative inquiry, ethnography, case studies, grounded theory, mixed-method research, action research or phenomenology. This research effort focussed on previous research findings and other researchers’ conclusions regarding the research problem and questions under investigation. While no traditional research instruments were used, the availability of previous research reports and academic writings, based on both qualitative and quantitative research, provided sufficient material to allow for an in-depth exploration of the literature.

### Data collection

This qualitative study used data obtained exclusively from secondary sources such as academic books, peer-reviewed journal articles, theses and dissertations, conference papers, research reports, government publications, newspaper articles and previous international and local studies with a similar focus. South Africa served as the geographical focus of the research effort, but international trends, opinions and theories were incorporated to ensure a comprehensive picture of the problem. To gain an understanding of the phenomenon of false information, other secondary sources such as mainstream websites, social media platforms and newspaper articles were also explored. To adhere to the requirement for relevance, the publication date of sources serving as illustrations of the dissemination of false information regarding COVID-19 was limited to the period 01 December 2019 to 05 March 2021. In order to prevent a haphazard and unstructured research effort, a conscious decision was taken to limit the study to the first year of the pandemic in South Africa. While this may be seen as a limitation to the study, an indeterminate timespan while the pandemic was still raging could have resulted in an open-ended effort with no clear conclusion. Finally, online resources dealing with predatory journals were consulted regularly to avoid making use of journals that would prove to be unreliable or inappropriate.

### Data analysis

The data collected while conducting the literature review required analysis to ensure its relevance and reliability, as well as to meaningfully describe and interpret it. Consequently, the focus was on false information, false information specific to South Africa, types of false information, the identities and motivations of propagators of false information, the reasons why people are susceptible to false information and the effect of false information on the social contract. A total of 130 accredited sources that relate to the scope of this study were analysed. To ensure the credibility of these sources, the following criteria were applied:

- Sources were selected based on their direct relevance to the research questions and objectives. Only sources that provided significant insights into the dissemination and impact of false information during the COVID-19 pandemic were included.
- The publication date of sources was crucial to ensure the information was up to date. Only sources published between 01 December 2019 and 05 March 2021 were considered to maintain the study’s temporal relevance.
- The credibility of the sources was evaluated based on the authors’ qualifications, expertise and affiliation with reputable institutions. Only works by authors with established credentials in relevant fields were included.
- Priority was given to peer-reviewed journal articles and academic books from reputable publishers, ensuring the sources had undergone rigorous review processes.
- Data were sourced from trusted academic platforms like JSTOR, Taylor & Francis Online, SAGE Journals Online, Google Scholar and institutional repositories to ensure high-quality and credible information.

By rigorously applying these criteria, the study ensured that only reliable and valid sources were included, thereby enhancing the transparency and credibility of the research methodology. Comparisons among different sources helped determine the prevalence of false information dissemination in South Africa during the first year of the COVID-19 pandemic.

### Theoretical background to the concept of false information

The phenomenon of false information has been part of human communication since at least 44 BC when the Roman Empire saw Octavian launch an unsuccessful smear campaign against Mark Antony using slogans imprinted on coins. Approximately 1500 years later, the invention of the Gutenberg printing press in the 15th century contributed considerably to the dissemination of false information. Del Vicario et al. (2016) argue that the World Wide Web is changing how people receive information, deliberate issues and form opinions. The WEF had included massive digital disinformation as one of the greatest risks to humanity as far back as 2013 (World Economic Forum 2013). Anderson (2021:1) asserts that academic interest in and research into false information are just as ‘historically embedded’ as the phenomenon itself. Barclay (2018b) points out that information is ‘a product of human thought and human effort’, and for this reason, it is rarely uncomplicated and never perfect. Kumar and Shah (2018) concede that there is a significant difference between false information and real information, but Barclay (2018b) cautions that the mere categorisation of ‘good’ information versus ‘bad’ information oversimplifies the issue.

Theoretical discussions on misinformation, disinformation and information disorder provide critical insights into the nature of false information. Misinformation generally refers to false or misleading information shared without harmful intent, whereas disinformation involves the deliberate creation and dissemination of false information to deceive and manipulate public opinion (Wardle & Derakhshan 2018). Additionally, the concept of information disorder encompasses misinformation, disinformation and malinformation, with malinformation describing factual information used maliciously to cause harm (Wardle 2018b). Understanding these distinctions is crucial for comprehensively addressing the spread and impact of false information.

The taxonomy related to information that is false, untrue, incorrect or imaginary is a potential pitfall in the study of false information. Pomeranz and Schwid (2021), Baines and Elliott (2020) and Wu et al. (2019) use misinformation as an umbrella term for ‘all inaccurate or false information or information of unknown accuracy, transmitted through any means’. However, following extensive research into the taxonomy of information types, Baines and Elliott (2020) find that the term misinformation as an overarching label is confusing and should be avoided. They contend that misinformation and disinformation cannot be used interchangeably as the spreading of false information is unintentional when it comes to the former and deliberate when pertaining to the latter. They emphasise the importance of unambiguous and scientific definitions being found for different information types.

This article uses the term false information to refer to fabricated and baseless information. Pomeranz and Schwid (2021:4) define false information as ‘information presented as fact that has been disproven as inaccurate or not truthful’. Other definitions of false information are difficult to find in the literature, as the concept is logical and obvious: if the information is not true, it must be false.

**FIGURE 1:**
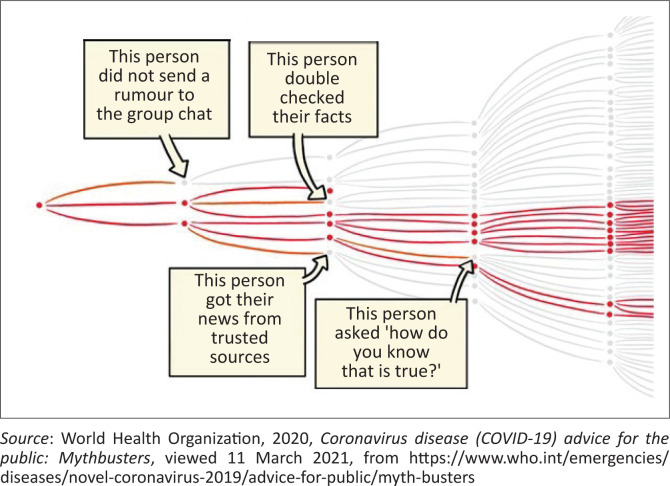
Schematic from the World Health Organization illustrating how misinformation spreads.

Vosoughi, Roy and Aral (2018) conducted a study of 126 000 news stories on Twitter between 2006 and 2017 and concluded that false stories have a 70% greater likelihood to be retweeted than true stories and that humans are more likely to forward falsehoods than automated robots. Barclay (2018b) points to the increase in the number of platforms used for communication as one of the reasons for information overload and the consequent surge in false information. South Africa, as an active participant in the global social media revolution, is almost certainly suffering its quota of information disorder. South Africa had approximately 30.1 million social platform users in 2021, and this figure is projected to increase to around 40.77 million by 2026. Against a January 2022 population estimate of just over 60.5 million, South Africa is positioned to experience a similar rate of false-information dissemination than is being experienced globally. False information is spread through propagation and susceptibility, but the World Health Organization (WHO) provides a schematic illustration of the process in Figure 1.

The WHO’s depiction of people who do not forward rumours, who use trustworthy sources, who double-check the facts when they are uncertain and who question the truthfulness of doubtful information suggests the need for judgement and prudence when information is received. This comes down to what is variably referred to as information literacy (Barclay 2018a), digital or news literacy (Ting & Song 2017; Waisbord 2018) or media literacy (Livingstone 2004; Molina et al. 2021; Wasserman & Madrid-Morales 2018). Livingstone (2004:5) asserts that literacy in general amounts to the ‘ability to access, analyse, evaluate, and create messages in a variety of forms’. Scheufele and Krause (2019) argue that the most challenging of these in terms of false information is the ability to evaluate, as those with a limited ability to evaluate struggle to determine the veracity of information.

### Types of false information

**FIGURE 2:**
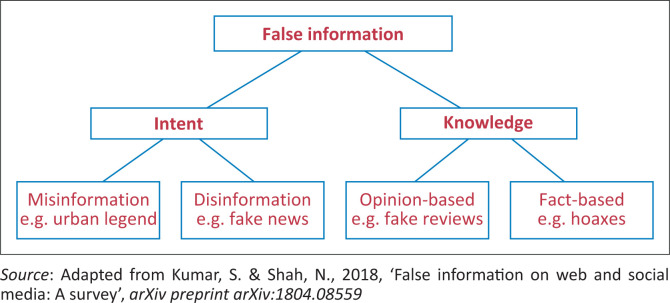
Categorisation of false information based alternately on intent and knowledge.

Kumar and Shah (2018) categorise false information into misinformation and disinformation, based on a person’s intent with the false information. In addition, Kumar and Shah (2018) posit a second classification of false information based on knowledge, where opinion-based false information does not involve a basic truth and fact-based false information relates to outright lies about subjects that have inherent truth. Consequently, Kumar and Shah (2018) presented the dual categorisation of false information (Figure 2) based on the intent with and knowledge of the information being created or disseminated.

**FIGURE 3:**
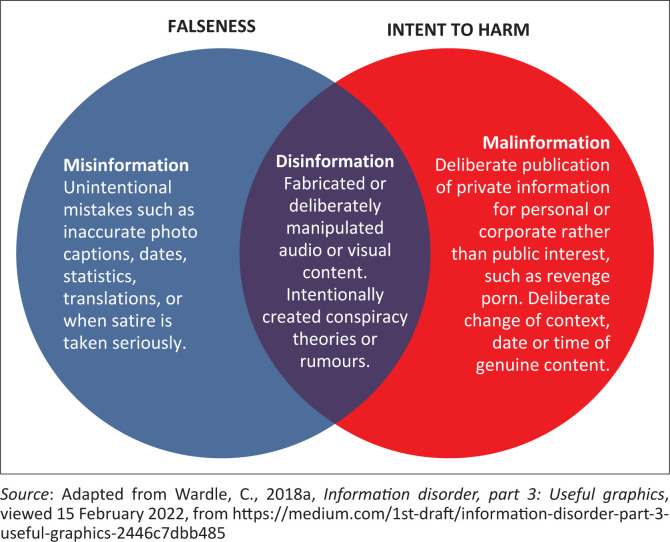
Types of information disorder.

Baines and Elliott (2020) tentatively describe three types of false information, namely, misinformation, disinformation and malinformation. These terms are differentiated based on the intent behind the spreading of the information: misinformation being unintentional, disinformation being deliberately misleading, and malinformation being factually reconstructed. The Aspen Institute (2021) holds similar views on the different types of false information in circulation and cites Dr Claire Wardle as the architect of the relatively new concept of information disorder. Wardle (2018a) approached the difference between misinformation and disinformation from a slightly different angle, asserting that in the case of misinformation, the person propagating the false information believes it to be true, while in the case of disinformation, the person is perfectly aware that the information is false. Malinformation is authentic but used to cause harm to an individual, an organisation or a country. Information disorder, represented by its three components, is illustrated graphically in Figure 3.

Fake news is a form of false information that has gained a strong foothold in societal discourse since 2016, but it is not new. Examples of propaganda, hoaxes, alternative facts and other forms of mischievous or malicious false information have littered human history. Turcilo and Obrenovic (2020) declare that misinformation, disinformation and malinformation are all forms of fake news. Barclay (2018b) describes fake news as any information that is purposefully produced with the claim of being true when, in fact, it is not and states that these assertions are problematic, as all forms of news can be classified as information, but not all information can be categorised as news.

Waisbord (2018:1866) describes the modern understanding of fake news to be ‘content featuring the style of conventional news intended to deliberately misinform’, thereby confining the definition to the realm of news. Allcott and Gentzkow (2017:213) follow the same reasoning, limiting fake news to ‘news [emphasis added] articles that are intentionally and verifiably false, and could mislead readers’. Allen et al. (2020) found that between 2017 and the final submission of their article in 2020, Google Scholar had reflected 2210 English-language articles with the words ‘fake news’ in their titles, while only 73 such articles had appeared between the launch of the platform in 2004 and the end of 2016. Allen et al. (2020) found that academic interest in online or social media-related sources of fake news outstripped academic interest in television-generated fake news by a considerable margin. Barclay (2018a) observed that fake news has transformed social media from a mostly harmless, if self-indulgent, way to communicate with friends and family into a high-capacity conduit for false or misleading information that, before the digital age, would have seen little or no circulation.

The proliferation of false information has been linked to the rise of predatory journals (Elmore & Weston 2020), which are supposedly academic and scientific journals that will ‘print’ anything in return for payment (Beall 2021; Moher et al. 2017). Jeffrey Beall started a blog in 2012 to publicise the names of publishers and stand-alone journals that were deemed to exhibit predatory tendencies (Beall 2021). Cabells International started publishing the Journal Blacklist (Linacre & Bisaccio 2023), whose list of predatory journals had surpassed 15 000 by September 2021. Additionally, the Directory of Open Access Journals (DOAJ) and the Think.Check.Submit blogs are online resources that help scholars to separate fact from fiction in their academic work (DOAJ 2022; Think.Check.Submit. 2022). The surge in false information has given rise to an increased impetus to find the truth, but the line between truth and fiction seems to be becoming increasingly blurred. The different types of false information are closely linked with the motivation behind the creation of false information and people’s susceptibility to false information.

### The propagators of false information

The dissemination of false information has significantly increased because of complex cultural and societal changes, such as the decline of trust in government entities, community institutions and journalism. This has been exacerbated by the polarisation and acrimony that permeate public discourse and the widespread distrust between individuals and powerful institutions. However, power and greed seem to have remained the core motivating factors behind the spread of false information. This view is supported by several authors who agree that the incentive to create and distribute false information can be divided into two main categories: ideological persuasion and financial gain. In the first instance, the motivation to spread false information has a strong political flavour, with agents involved in this practice seeking to advance specific political points of view and using misleading facts and data within their articles.

Elsamni (2020) elaborates on this point, stating that some countries target the national security of their opponents, while at the intrastate level, political parties discredit the agendas of their rivals. The 2016 United States presidential election and its aftermath are widely cited as a possible trigger for the current spike in false information. Other examples of the use of false information in a political context include the mudslinging that characterised the exit of the United Kingdom from the European Union, the presence of weapons of mass destruction and the alleged threat of terrorism used to justify the George W. Bush administration’s 2003 incursion into Iraq, and ongoing disinformation campaigns between the United States and Russia and China, respectively. False information also targets other spheres of society, such as the debates around climate change, abortion, childhood vaccination and capital punishment. Waisbord (2018) asserts that fake news has been politically weaponised.

The employment of false information for financial gain is known as advertising. This practice involves ‘clickbait’ headlines, where users are enticed to click on links that take them to commercial sites. These sites masquerade as news and have contributed significantly to distrust of the news media and scepticism towards online information. While these hoax sites can be very lucrative, they are mercenary in nature, as the goal is to make money and not to promote the agenda of any political group, organisation or commercial venture. Manoim and Mare (2017) caution that individuals who get caught by once-off hoaxes should be distinguished from the systematic ensnarement of the public through the publication of false information claiming to be factual for political, economic or cultural benefit.

The intention of the progenitor of content is different in each case, and Wardle and Derakhshan (2018) posit that information disorder originates in three phases: creation, production and distribution. The mastermind or agent behind the creation and publication of false information can determine its extent and impact. High levels of networking and automation may also exacerbate the effect. Additionally, if one agent is responsible for the creation of a false message, another for its production, and yet another for its initial distribution, the origins of the deception may become difficult to trace and the scale of the impact may become greater. It is therefore important to assess and thoroughly understand the roles played by the agents of false information.

Wardle (2017) posits eight ‘Ps’ (poor journalism, parody, to provoke or ‘punk’, passion, partisanship, profit, political influence or power, and propaganda) as motivation for the creation of false information. These categories of motivation can be measured against different types of content created for specific purposes, such as satire, false connection or misleading content. Muigai (2019) argues that false information lies on a scale of intent ranging from humour and mischief to malice. Using the eight ‘Ps’ of Wardle (2017) as a yardstick, Muigai (2019) advances eight reasons (Table 1) for the creation of fake news (false information).

**TABLE 1:** Eight reasons for the creation of fake news (false information).

No.	Reasons for creating fake news
1.	To deliberately mislead to damage a group, entity or person.
2.	To secure financial gain by enticing people to click on sites that run advertising (clickbaiting).
3.	To influence people into taking a stance in support of or in opposition to a cause or political candidate.
4.	To trick or prank people.
5.	To increase the popularity of social media platforms (such as the Facebook newsfeed).
6.	To improve readership rates through appealing headlines on social media that entice people into sharing content without evaluating or even reading it.
7.	To exploit information bias – people are more apt to entertain content that reinforces their beliefs and discard content that does not.
8.	To polarise political discourse and propagate hostility among government actors, especially during elections.

*Source:* Muigai, J.W.W., 2019. ‘Understanding Fake News’, *International Journal of Scientific and Research Publications (IJSRP)*, 9(1), 29–38. https://doi.org/10.29322/ijsrp.9.01.2019.p8505

### Public susceptibility to false information

Recent research highlights how easily people can be swayed by false information (Ecker et al. 2022; Pennycook & Rand 2021). Prior to 2004, studies on this topic were mostly analytical with limited empirical data (Flynn, Nyhan & Reifler 2017). Table 2 encapsulates the key findings and observations from various sources on public susceptibility to false information, especially in the digital age.

**TABLE 2:** Summary of key research findings on public susceptibility to misinformation.

Researchers and authors	Key findings and observations
Lewandowsky et al. (2012)	Misleading info often lacks warnings. Once false information is internalised, rectifying it is challenging because of factors like source reliability.
Del Vicario et al. (2016)	Individuals form communities based on shared interests leading to confirmation biases and polarisation.
Lazer et al. (2017)	Emphasised the role of social processes in misinformation acceptance, such as source credibility and information-seeking biases.
Ting and Song (2017)	People lean towards information aligning with their beliefs. Frequently encountered and negatively charged false information spreads efficiently.
Barclay (2018b)	Emphasised digital literacy needs. Warned of the ‘information-bubble phenomenon’ and how emotions can drive false narratives. Introduced the term ‘glurge’ for feel-good misinformation.
Chakrabarti, Stengel and Solanki (2018)	In the digital era, individuals use coping strategies like selective consumption. However, verification techniques remain limited and sometimes ineffective.
Ireton and Posetti (2018)	Innate human biases play a role in hindering the acceptance of new truths.
Kumar and Shah (2018)	Discerning truth from fiction is tough for humans, especially when misinformation is compelling. ‘Echo chambers’ in recommendation algorithms can exacerbate false beliefs.
Mavridis (2018)	Used a minor sample from the Ellinika Hoaxes Facebook^[1]^ group using the Uses and Gratifications Theory^[2]^ to study reactions to misinformation, emphasising the need for broader quantitative research.
Pangrazio (2018)	Noted two online reading changes: increased reliance on social networks over news sources and the evolution of news consumption as a social venture. Introduced the term ‘homophily’ to describe selective interaction with like-minded individuals.
Nemr and Gangware (2019)	Delved into psychological factors increasing susceptibility to misinformation, such as the desire for social identity, cognitive overload and ‘belief perseverance’.
Rennen et al. (2020)	Fact-checking of English-language info rose by 900% in early 2020.
Vicol (2020)	Highlighted three cognitive biases influencing beliefs and noted that age and education play roles in discerning fact from fiction. Older individuals are more resistant to changing beliefs.
The Aspen Institute (2021)	Emphasised that misinformation is not the root of all societal issues.
Beauvais (2022)	Suggested susceptibility to fake news hinges on media influences and individual psychological and sociological factors. Key reasons include intelligence, bias, trust and stress.
Edelman Trust Barometer (2023)	Revealed a ‘cycle of distrust’, with 76% of global citizens concerned about fake news. Trust in various institutions, including government and media, has declined.

In essence, individuals are susceptible to misinformation because of cognitive biases, societal influences, digital media’s structure and emotional manipulation. While the phenomenon of false information spread via social media is still relatively new, research in this regard is reasonably reliable, although there may be a need for more intensive quantitative research, specifically relating to the manifestation and impact of false information in regions outside the United States and Europe.

### Allowing the government to govern: The impact of false information on the social contract

The concept of the social contract, which traces its roots to thinkers such as Hobbes, Locke and Rousseau (Morris 2000), suggests that individuals sacrifice some natural rights to obtain societal benefits and security. Central to this idea is the mutual agreement between individuals and the governing entity. Moon (2013) interprets this as lending legitimacy to governments, with Fabre (2013) emphasising that this legitimacy depends on the consent of the governed. However, there is no universal agreement on the specifics. For instance, Shaapera (2015) highlights that while Hobbes saw the contract as an agreement between the people and their rulers, both Locke and Rousseau viewed it as a pact among the citizens themselves for mutual security.

As societies evolved, so did the notion of the social contract. Particularly, the emergence of property rights altered its framework. Central to this modern understanding of the social contract is the role of information. Fukuyama (2013) underscores the importance of transparency and effective communication in governance, emphasising that it is crucial for citizens to be informed. But in today’s digital age, misinformation poses a significant challenge. Bölükbaşi and Mohammed (2020) shed light on the negative impacts of false information on democracies, despite the technological advancements that have revolutionised communication. The Berggruen Institute (2023) similarly asserts that the digital age necessitates new solutions to these unique challenges.

The COVID-19 pandemic intensified these challenges (Cinelli et al. 2020; Hosseinzadeh et al. 2022). Razavi et al. (2020) indicate that many nations grappled with the pandemic on the backdrop of a weakened social contract. Clark (2020) expands on this, suggesting that the pandemic exposed flaws in the existing social contract, with governments demanding more from citizens than the benefits they offer in return. This crisis, he believes, may give rise to new societal arrangements and contracts. Emphasising the gravity of misinformation during the pandemic, the World Health Organization took proactive steps, launching a ‘Mythbusters’ page to dispel COVID-19-related falsehoods (World Health Organization 2023).

## Research findings

### Actors and motivations behind the dissemination of false information

The complexity of false information dissemination necessitates an exploration of both its propagators and their motivations. The landscape of misinformation is vast, and no singular profile fits the bill of a typical disseminator. From politicians and rival nations manipulating narratives to ordinary individuals equipped with digital tools, the modern era has democratised the ability to spread falsehoods.

Concrete examples illustrate the diversity of these actors. For instance, during the 2016 US presidential election, Russian operatives utilised social media platforms to disseminate disinformation aimed at influencing voter behaviour and sowing discord (Mueller III 2019). Similarly, in the context of the COVID-19 pandemic, various conspiracy theories about the virus’s origin and the efficacy of vaccines were propagated by influential figures such as celebrities and political leaders, which further fuelled public scepticism (Freeman et al. 2022). In South Africa, false claims regarding COVID-19 cures and government responses were often spread by local influencers and community leaders, exacerbating public fear and mistrust (Geldsetzer 2020).

Yet, understanding the motivations may be more insightful than merely identifying the actors. Predominantly, the pursuit of power, political ideologies and financial incentives emerge as compelling reasons. Wardle (2017:1) encapsulates this in the eight ‘Ps’, which, besides political and profit motives, highlight varied reasons like incompetence (poor journalism), comedic intents (parody), emotional triggers (passion) and deliberate deceit (propaganda). Building on this, the concept of ‘uncritical publics’, as introduced by Ireton and Posetti (2018:15), underlines the vulnerability of an uninformed populace. This vulnerability, when paired with advanced communication technologies, offers misinformation agents a potent medium to manipulate narratives globally. For example, during the Brexit referendum, political entities used targeted misinformation campaigns to influence public opinion on key issues such as immigration and economic policy (Howard & Kollanyi 2016). Financial incentives also drive the proliferation of false information. Fake news websites often rely on sensationalist headlines to generate clicks and ad revenue, a practice seen widely during the COVID-19 pandemic when numerous sites promoted unverified treatments for the virus (Pennycook et al. 2020). Additionally, actors such as anti-vaccine advocates exploit emotional triggers by sharing alarming and often false stories about vaccine side effects to sway public sentiment (Loomba et al. 2021).

These examples illustrate how various actors exploit different motivations to disseminate false information, highlighting the complexity and multifaceted nature of this phenomenon. Detailed examples of these motivations in action can be seen globally. For instance, during the 2014 Ukraine crisis, Russian state-sponsored media spread disinformation to justify the annexation of Crimea and to destabilise Ukrainian political structures (Helmus et al. 2018). The tactics employed included the creation of fake social media profiles, the spread of fabricated news stories through state-controlled outlets, and the use of bots to amplify these messages. Similarly, in Myanmar, military forces used social media platforms to incite violence against the Rohingya minority by spreading false reports of Rohingya attacks on Buddhists, thus fuelling ethnic tensions and violence (Mozur 2018). Financially motivated actors include websites that capitalised on the COVID-19 pandemic by selling fake cures, such as colloidal silver, which was promoted as a treatment despite no scientific evidence supporting its efficacy. These websites used aggressive marketing techniques and fear-based advertising to attract vulnerable individuals seeking protection from the virus (Bruns et al. 2020).

### Navigating the terminologies of false information

The lexicon of false information is itself a contested arena. While intent is universally recognised as a key determinant in classifying misinformation, there is no consensus on a single taxonomy. Some scholars limit their descriptors to ‘misinformation’ and ‘disinformation’, while others like Baines and Elliott (2020) and Wardle and Derakhshan (2018) introduce terms like ‘malinformation’. The term ‘fake news’ also finds prominence in some discourses as a catch-all phrase.

In this context, ‘malinformation’ stands out because of its inherent malicious intent. Elaborating further, Wardle and Derakhshan (2018) discern misinformation as false narratives shared by those who believe them, whereas disinformation is spread by those cognisant of its untruth. With technological advancements, the ease of spreading such falsehoods has surged, impacting even academic spheres as evidenced by the rise of predatory journals.

### The motives behind people’s willingness to entertain and forward potentially false information

A considerable amount of research has been done in recent times on the susceptibility of people to false information. The review of the literature has therefore allowed several findings to be made in this respect. These findings are shown in Table 3.

**TABLE 3:** The motives behind people’s willingness to entertain and forward potentially false information.

No.	Determinants of susceptibility to false information	Description
1.	Limited evaluation capacity	Owing to factors like information overload and deteriorating online reading skills, many individuals have trouble distinguishing between true and false information. Particular demographics, notably the less educated and the elderly, tend to disseminate false information more frequently.
2.	Inherent biases	Cognitive biases such as confirmation bias, sender primacy and motivated reasoning lead many individuals to stick to false information even when corrected, perpetuating their existing views.
3.	Worldview influence	Personal worldviews drive selective information consumption, potentially leading to the creation of echo chambers and a heightened spread of false information.
4.	Societal pressures	The inherent need to fit in and remain updated within one’s social circle often prompts people to share unverified information. Societal issues like inequality amplify this trend.
5.	Emotional factors	According to Ting and Song (2017), certain neurological processes could predispose individuals to adopt and disseminate false information if it offers an emotional salve.
6.	Distrust in institutions	Decreased trust in major establishments such as the government, media and academia pushes individuals towards false narratives. They may even challenge peer-reviewed content.
7.	Reluctance to correct	Even when presented with validated truths, many individuals display an unwillingness to revise their false beliefs.

### The effect of false information on social cohesion and trust in government

The essence of the social contract is a mutual exchange: citizens grant certain rights to the government, expecting security and welfare in return. For this agreement to flourish, reliable communication is paramount. However, in the digital age, where governments increasingly use platforms like social media, false information poses a grave threat to this accord. Bölükbaşi and Mohammed (2020) stress the debilitating effects of false information on governments’ ability to uphold their side of the social contract. Drawing from the South African context, while the Constitution guarantees rights such as freedom of belief and expression, there is an implicit expectation that citizens reciprocate by responsibly handling and disseminating information.

### False information in South Africa in the first year of COVID-19

According to Geldsetzer (2020) and Baines and Elliott (2020), the nature and accuracy of information individuals receive significantly impact their perceptions and behaviours concerning the COVID-19 pandemic. While South Africa took steps to counteract COVID-19 misinformation, much of this false information originated beyond its borders. After detecting its first COVID-19 case on 05 March 2020, South Africa swiftly declared a national state of disaster, followed by a series of strict lockdowns.

To ensure adherence to these lockdowns, the South African Police Service (SAPS), assisted by 76 000 National Defence Force members, enforced stringent restrictions which included the closure of national borders and suspension of public transport. However, the strict measures and uncertainty surrounding them fuelled a plethora of rumours, conspiracy theories and falsehoods.

Staunton, Swanepoel and Labuschaigne (2020) discuss the South African government’s dilemma between prolonging the strict lockdown for public health reasons and easing it for economic concerns. While the initial lockdown effectively limited virus spread, the government’s decision-making process, which lacked extensive public engagement, fostered distrust and criticism. To counteract the misinformation, the government launched an extensive campaign and established a dedicated page on its website to debunk fake news.

Misinformation spread rapidly before and after the initiation of the hard lockdown. For instance, one individual was arrested for falsely claiming that COVID-19 tests spread the virus, although his case was eventually dismissed. The government leaned on Africa Check to monitor false ‘announcements’ about economic relief, recruitment drives and other topics, including misconceptions about COVID-19 and its treatments.

The South African government issued a media statement on 15 April 2020 warning of the consequences of spreading false information regarding COVID-19. This statement included a hi-tech monitoring and evaluation process to assess complaints and reports from the media, the public and other sectors of society, and the ability to take down fake news items on a range of platforms and submit cases to the SAPS for investigation and prosecution. The government also started a site called Fake news – Coronavirus COVID-19 on its official website early in the pandemic to enlighten people regarding false information, which goes against the tenets of the social contract between the government and the people and essentially represents a breach of contract on the part of the latter.

In a November 2020 retrospection of the impact of false information on South Africa in the first 10 months of the COVID-19 pandemic, the Scientists Collective (2020) reports that the internet and media have been ablaze with stories, reports and ever-changing guidelines on how to stay safe in a world that appears to be out of control. They point out how difficult it is to distinguish between the truth and the myriad of false information and myths, especially while learning how to deal with COVID-19 in real time. To educate the public, they debunk several false claims that made the rounds in South Africa from the start of the pandemic. These are included in Table 4.

**TABLE 4:** False claims that are unsubstantiated assertions.

No.	Prominent false claims in South Africa from the start of the pandemic
1.	‘COVID-19 is a scam and there is no virus’.
2.	‘Doctors write Covid as the cause of death if someone has tested positive, no matter what they die of, whether it is cancer or a car accident, with COVID-19 massively over-reported’.
3.	‘The World Health Organization was created by people like the Rockefeller or Gates families to control global health policy’.
4.	‘The virus (SARS-CoV-2) which causes COVID-19 hasn’t been isolated in South Africa’.
5.	‘The SARS-CoV-2 virus was created in a Chinese laboratory (or by the CIA or the Russians)’.
6.	‘COVID-19 is no more dangerous than the flu and it is crazy to worry about a disease that is more than 99% survivable’.
7.	‘Mask-wearing is controversial’.
8.	‘The vaccines are just a money-making scheme and are a way to track you and/or collude with 5G networks’.
9.	‘Fogging and/or deep-cleansing and/or mouthwash (insert your favourite intervention here) will save you’.

*Source:* The Scientists Collective, 2020, *Fake news and misinformation kill: How can you trust what you are told about Covid-19?* viewed 08 February 2022, from https://www.dailymaverick.co.za/article/2020-11-22-fake-news-and-misinformation-kills-how-can-you-trust-what-you-are-told-about-covid-19/?utm_medium=email&utm_campaign=The%20Scientists%20Collective%20Special%20Edition%203%20December%202020&utm_content=The

COVID-19, coronavirus disease 2019; SARS-CoV-2, severe acute respiratory syndrome coronavirus 2.

They debunk several false claims that made the rounds in South Africa from the start of the pandemic, showing the widespread and disruptive influence of false information on the South African government’s attempts to address the pandemic and the impact on and complicity of the South African public. The South African government embarked on a wide-ranging information campaign and criminalised the dissemination of false information concerning the pandemic and the government’s fight against it, showing that false information was prevalent in South Africa in the first year of the pandemic. The scourge of false information can arguably be blamed on distrust in government and cynicism towards the efforts of international health authorities, but the finding is that false information was very prevalent in South Africa during the first year of the COVID-19 pandemic.

This study goes beyond existing literature to focus on the challenges that the South African government faced regarding misinformation during the pandemic. It highlights the intricate dynamics between foreign misinformation sources, the quick spread of false claims and South Africa’s countermeasures. While other research broadly addresses governmental responses, this work provides an in-depth analysis of South Africa’s strategies, from lockdowns and military aid to combating disinformation and introducing dedicated online platforms. By detailing specific false claims, the study offers a detailed view of the misinformation challenges faced by South Africa, presenting fresh perspectives on a nation navigating a pandemic amid rampant disinformation.

## Discussion and conclusions

This research effort sought to describe the phenomenon of false information, to determine its prevalence in South Africa during the first year of the COVID-19 pandemic, and to establish whether it influenced the social contract between government and citizens. The research design involved a qualitative review of available secondary sources dealing with the different sub-topics delineated by the research questions. The review of the literature found that there has been an increase in the dissemination of false information globally since at least 2016. This trend has continued as the global COVID-19 pandemic evolves, and South Africa has seen its fair share of false information relating to COVID-19. The country is just as much a participant in the digital age as most developing countries and can compete with most developed countries in this regard. It is therefore not surprising that South Africa is confronted with similar levels of false information as most other nations. The findings described in this article make it possible to conclude that there was a high prevalence of false information in South Africa during the COVID-19 pandemic and that it placed a strain on the South African government’s efforts to govern during a time of crisis. The findings can be extrapolated to most other countries in the world, especially those that espouse democracy and advocate freedom of speech. False information, when it is disseminated with harmful or seditious intent, is dangerous and can threaten the social order and the social contract.

This article suggests that recommendations can be made at four levels: government, ordinary citizens, academia and the media. The South African government needs to take cognisance of the increasing polarisation brought about by systemic problems such as inequality, poverty and unemployment and be sensitive in their approach to sharing information. Aggressive campaigns that foster national identity and a sense of patriotism may alleviate some of the issues in this regard. The South African government should also acknowledge the decline in public trust towards them and employ measures to regain that trust. Government decision-makers and spokespersons who are deemed to be competent and who elicit support must be at the forefront of government communication.

The most important details in this text are the measures that can be taken to reduce the dissemination of false information. These include broader, more frequent communication with the public in times of crisis, and the delivery of that communication by respected representatives of a wide range of organisations with proven track records. It is also important for the government to follow through on prosecutions and punitive measures to avoid their policies being viewed as empty threats. Ordinary citizens have become a significant threat to the government when it comes to false information, but it could be that their dissemination of false information has more to do with ignorance than malice. People need to become more open to views that do not correspond with their own, even if it is only to broaden their knowledge and provide themselves with more options. Ignorance or limited evaluation skills when it comes to new information will also hamper the effective evaluation of that information.

In the case of academia, controls that ensure academic integrity and reliable research must be maintained and possibly improved. These measures include adherence to strict ethical codes and rigorous peer review. Predatory journals must be exposed for what they are, and in this regard available online and other resources that pinpoint dishonest, self-serving and fly-by-night ‘scholarly’ publications and publishers must be fully utilised. The academic community would also do well to find ways to establish broader access to research findings and scholarly texts to improve knowledge among the population and instil trust in academic institutions.

The media also has a hand in the dissemination of false information and a responsibility to combat it. Despite the arrival of social media and the rise of the citizen journalist, the so-called ‘Fourth Estate’ continues to play a significant role in the circulation of news and government messages, and the media, therefore, needs to maintain its integrity and objectivity in its delivery of information. Harmful and reckless journalism should not be allowed to thrive without consequences.

When contemplating future research on the impact of false information on the social contract, especially in times of crisis, one must acknowledge the relative infancy of the phenomenon of digital false information. Although much has been written on this topic, new knowledge is being accumulated almost every day – a considerable amount has already been added to the literature after this article’s cut-off date of 05 March 2021. The possibilities for future research are therefore wide, and hopefully, this research effort has at least laid some groundwork for such endeavours.

The ever-evolving phenomenon of false information requires more empirical research to understand its prevalence and impact. Other topics that may merit further research include the contested taxonomy of the false information phenomenon, the effect of conspiracy theories on societies and governments, and the rise of fact-checking and information verification as a non-negotiable tool in the modern field of communication. Government reactions to the pandemic and the unprecedented restrictions imposed on the freedoms of citizens may have changed the essence of the social contract as we know it.
